# Supplementary material for: Patterns of Intron Gain and Loss in Fungi
Source: PLoS Biol. 2004 Nov 30;2(12):e422. doi: 10.1371/journal.pbio.0020422 (PMC532390; doi:10.1371/journal.pbio.0020422)
Supplement: Table S1 — Also available at http://genes.mit.edu/NielsenEtAl/. (4.3 MB ZIP). [file pbio.0020422.st001.zip › NielsenEtAl/html/878.html]

AN0072.1.NCU04153.1.MG08626.1.FG07326.1


```
 CLUSTAL W (1.82) Multiple Sequence Alignments - Introns Inserted


Sequence 1: NCU04153.1	478 aa
Sequence 2: MG08626.1	497 aa
Sequence 3: FG07326.1	494 aa
Sequence 4: AN0072.1	392 aa
Alignment Length: 525 aa
Number Identitical Residues: 136 aa
Alignment Score (without introns) 7755


MG08626.1 	MVIPSVTGLDGIFA1INKPMGMSSAQVIRDCQAYFQPSRLFAPMLAREAEEARRDAATQG
NCU04153.1	MAQNSLA--EGVFA1INKPYGMSSAQVIRDCQHHFNPSKLFAPLLAADRALREKEGRYQ-
FG07326.1 	MATGIIR--EGVFA1INKPCGQSSAQVIRECQQVFNPSEFFKPLIQSEVAKRMNENSGE-
AN0072.1  	MSGEKIY--EGVFA1VHKPQGVTSADVIRKLQHHFNPSELFRPWLETERAKRNRESNFQ-
          	*    :   :*:** ::** * :**:***. *  *:**.:* * :  :     .:   : 

MG08626.1 	KHNRKWKKRKQDPRVKMGHGGTLDPLATGVLILGVGRATKSLQNFLECTKTYETIVVFGA
NCU04153.1	KQRRTHAKREIN--VKIGHGGTLDPLATGVLILGVGKGTKALHDFLGCTKTYETVVVFGA
FG07326.1 	SARRKALKRASQ--VKIGHGGTLDPLATGVLILGIGCATKALPQFLECTKTYETTIVFGA
AN0072.1  	--RRRRRSQRLD--VKIGHGGTLDPLATGVLVTGVGKGTKFLNDFLGCTKTYETVVIFGA
          	  .*   .:  :  **:**************: *:* .** * :** ******* ::***

MG08626.1 	ATDTYDRVGRILAKKSYDEITRDKVEKQLDAFRGKYKQMPPLYSALKMNGKPLYEYAREG
NCU04153.1	STDTYDRVGKLIKKGDYSTITREQVEEALNSFRGKFQQMPPLYSALKMNGKPLYEYAREG
FG07326.1 	ATDSYDRVGRILTKRPYEHITRELVEKEIASFKGRQIQIPPLFSALKMNGKPLYEYAREG
AN0072.1  	ETDSYDRLGKIVRRAPYEHITREAVEKALEQFRGKIMQRPPIFSALKVQGKKLYEYAREG
          	 **:***:*::: :  *. ***: **: :  *:*:  * **::****::** ********

MG08626.1 	KPIPREIETREVEVLGLELLEWYEPGTHNHRWPTEEAGIAEKSLAESVWRVEHEQATG--
NCU04153.1	KPIPREIETREVDVTELELTEWYEPGTHNHRWPTEEAEQAEKNVVKSVWQVAKNQQDGTT
FG07326.1 	KPIPRQIEGREVDVKEIEMVEWYEPGEHNHRWPTEEAEAAERNLAEQVWRVKKQQESN--
AN0072.1  	KEPPIEIQSRPVEVTDLRIVEWYEPGTHEYTWPEEEAPADERAVAEKLLAKDDS------
          	*  * :*: * *:*  :.: ****** *:: ** ***   *: :.:.:    ..      

MG08626.1 	-----KKLTPEQGAEEDRAFAAHQSFKEQAEERQDKLVFERGQKRKAEESNGRPNKKQQG
NCU04153.1	STEEAATLIPEVVEKEVKALANHESNKRAAEERVDELVSE--------EPAGKKRKTETG
FG07326.1 	-----KPLSPEEKQQDDKAIAAHETFKRNFEERQDALIKD--------APSKKSRKSKEP
AN0072.1  	-----LPIVP--AADDADGSSKRKSPPAEGSEGNETTASN--------AAAKKQKVSAEG
          	       : *    .:  . : :::     .*  :    :         .  : . .   

MG08626.1 	KQGREQLMSGALG-----ELPPGGPSKGRGSDLVPPAPSADTPPPWDGKGPPACKIRMTV
NCU04153.1	--AAEPMMSGALKDKKGKDKKQDKYAKNRGLDLAPPPPSPDTPFPWEGKGPPAAKIRMTV
FG07326.1 	-----RMMSGALG-----KLPQPTYS-TKGQNLVPDAPDSSTPPPWSDQGPPACKVRLTV
AN0072.1  	-------EAASAP-----EQTKAPEAVEEATTKTKPESAEEMKPLAP---PPAVKITMTV
          	        :.:       .      :  ..   .   .  .         *** *: :**

MG08626.1 	TSGFYVRSFAHELGEKVGSAALMAELVRNRQGDFVLEGDNCLEYDDLVKGEDAWGPKLEK
NCU04153.1	TSGFYVRSLCHDLGEKLGCGAMMAELVRSRQGEFVLGTDNCMEYSDLALGEEVWAPKIQK
FG07326.1 	TSGFYVRSFCHDLGAKLDSAGLMAELSRTRQNHFEVGSENCLEYGDLAKGENVWGPKVAG
AN0072.1  	SSGFYVRSLAHDLGKALGSCGLMSALVRSRQADYTLEPDKILEYKDLDAGEEVWGPKVKG
          	:*******:.*:**  :.. .:*: * *.** .: :  :: :** **  **:.*.**:  

MG08626.1 	VLHQWSS--------------THLQGVGPLKS--VYAEEPKTTAVNQEGPSGTAADKKPA
NCU04153.1	ALDLWNN--------------KDKAGKAQAVEPVVKKEEVKTEEVKAEEIK--AEDDK--
FG07326.1 	MLKKWNEGLGAGPRGPAGPSEKSQKNETKRSGSPTRKQNKGEKRRRSESPNGAGSPRRKA
AN0072.1  	FLEEWER---------------------------KRAAEKRSN-----------------
          	 *. *.                                :                     

MG08626.1 	TDEKPAAASVEESQTKESGVKAEETKPTDTKGETIDGYEEWNGFSD
NCU04153.1	TEKQPTPTPTPEQPTNEVNVEESASAKDGAKA--------------
FG07326.1 	AALESKQDSKPVKEPMKEPVKEAKEEATPDAGNRSDDEKSWNGIED
AN0072.1  	----------------------------------------------
          	
```
